# Biocompatibility and Oxidative Stress Profiling of Laccase-Catalyzed Conversion Products of Biomass-Derived Phenolics

**DOI:** 10.3390/toxics14070550

**Published:** 2026-06-24

**Authors:** Varun Chauhan, Salah-Ud-Din Khan, Mohsin Khan, Mohammed Sharique Ahmed Quadri, Anis Ahmad Chaudhary

**Affiliations:** 1 Government Institute of Medical Sciences, Greater Noida 201310, India; varunc1784@gmail.com; 2 Department of Biochemistry, College of Medicine, Imam Mohammad Ibn Saud Islamic University (IMSIU), Riyadh 11623, Saudi Arabia; 3 Department of Education, Government of Jharkhand, Ranchi 834006, India; 4 Department of Basic Medical Sciences, College of Medicine, Almaarefa University, Dariyah 13713, Saudi Arabia; 5 Department of Biology, College of Science, Imam Mohammad Ibn Saud Islamic University (IMSIU), Riyadh 11623, Saudi Arabia

**Keywords:** biomass-derived phenolics, catalase, ELISA, fungal laccase, malondialdehyde, oxidative stress, superoxide dismutase

## Abstract

The safety profile for bio-derived phenols post-oxidation and their related antioxidant/redox potential remain largely under-explored. Oxidation by fungi, in terms of environmental impacts via fungal oxidation by enzymes, remains an attractive strategy under milder conditions, since it is one route by which many naturally occurring lignocellulosic phenols are modified; thus, an immediate need still exists for characterizing the effects that these modified phenolic compounds may have. Methodology: We examined four different biomass-derived phenolics—vanillin, ferulic acid, syringaldehyde and guaiacol—that were oxidized with fungal laccase and characterized their effects on normal human lung fibroblasts and levels of cellular oxidative stress. Laccase activity was evaluated via the ABTS method and through simple observation and UV-Vis spectroscopic scanning of the phenolics in question, and compared with the untreated version of each phenolic. In addition to assessing the cytotoxic effect and oxidative stress generated by the phenols alone, an ELISA-based measurement assay was used to investigate the relative abundance of malondialdehyde (MDA), superoxide dismutase (SOD), catalase (CAT), glutathione peroxidase (GPx) and reduced glutathione (GSH) in the human normal lung fibroblast cell line under varying treatment regimes, complemented by phase-contrast microscopy. Scores integrating the biomarkers were analyzed via clustering, PCA, radar and Pearson correlation analyses, to discern distinct trends in antioxidant potential after laccase conversion. Observations: Each of the four tested phenolics demonstrated the presence of laccase activity, leading to substantial differences in visible appearance compared with the control and characteristic absorbance shifts at differing wavelengths from the original molecule. Cell viability dropped dramatically as phenol concentration was increased and the untreated phenolics resulted in diminished confluence and induced greater levels of oxidative damage, from guaiacol and syringaldehyde. Laccase treatment resulted in higher MTT reduction activity and improved cellular morphology compared with the corresponding untreated phenolic compounds. Untreated phenols induced the highest levels of MDA, while decreasing SOD, CAT, GPx and GSH levels. Post-oxidation with laccase, there were lower amounts of lipid peroxidation, along with improved levels of antioxidant activity compared with the control phenol. Multi-technique analyses show clear distinctness between the untreated and laccase-converted phenolic groups. Clustering with multivariate techniques separated all cell groups in line with control samples, grouping the laccase-converted treatments towards the middle and displaying an inverse relationship between MDA and the antioxidant markers. Conclusions: Laccase conversion markedly decreases the adverse effects that bio-derived phenols have on normal cell viability and induces fewer detrimental effects on the cellular redox balance. This is a critical discovery in terms of finding greener methods by which to upgrade bio-derived substances as we research these lignocellulosic phenols. By employing ELISA-based measurements along with multiple analysis techniques, we present a suitable paradigm for studying biological effects in all bio-based goods intended for pharmaceuticals, packaging materials, nutraceuticals or a host of different applications.

## 1. Introduction

It is not possible to switch entirely away from fossil-based chemistry because we require organic compounds. As one strategy to replace petroleum-based feedstocks and pollution with eco-friendly chemical processes, renewed attention has been given to renewable chemical products and enzymes that can catalyze value-added product manufacture using biomass-derived phenolic monomers. For instance, wood lignin—a widely available aromatic polymer derived from lignocellulosic biomass—is a precursor of various bio-products, such as phenolic compounds (e.g., vanillin, ferulic acid, syringaldehyde, guaiacol, p-coumaric acid, syringic acid) that are not only valuable green chemicals but are also involved in important activities related to cell antioxidant defense and protection against microbial and inflammation pathologies [1]. However, the wide variation in the solubility of these compounds in water, along with their intrinsic reactivity, dose-dependent toxicity in cells and scarcity of quantitative information on cellular oxidative effects, restricts their direct use in some promising biomedical applications or as food-contact/packaging materials, among others.

Laccases (multi-copper oxidoreductases) are biocatalysts that oxidize phenolic and other aromatic compounds, utilizing molecular oxygen as the final electron acceptor, while generating water and the oxidized substrate in return. These reactions are useful for green catalysis because no harsh and polluting reagents are necessary and many operate under favorable aqueous conditions and relatively mild temperature. Microbial (bacteria and fungi) and plant laccases are often explored for lignocellulosic biomass treatment and valorization, delignification of lignocellulosic residues, detoxification of phenolic-rich hydrolysates and conversion of some abundant bio-based phenolics (including those from lignin) to higher-value functional and bioactive products [2,3,4]. Through enzyme catalysis mediated by laccase, phenolic substrates become converted into reactive phenoxy radicals, as well as more complex oxidized products (e.g., quinones and polymeric compounds), altering some of the chemical properties (including water solubility, antioxidant potential) and influencing biological activity, potentially for the better. For example, the antioxidant properties of phenolic-rich extract from different fruit peels were increased after treatment with the laccase-mediated reaction due to increased formation of new reactive species. Hydroxytyrosol treated with fungal laccase demonstrated antioxidant properties and conversion into different derivatives [5,6]. Therefore, enzymatic conversion should be investigated as a strategy to obtain functional biomaterials/bioactive mixtures of improved quality and potentially more favorable safety profiles than a simple degradation mechanism that reduces quantity. Therefore, laccase-mediated catalysis offers several advantages over conventional chemical oxidation methods, including operation under mild aqueous conditions, utilization of molecular oxygen as the terminal electron acceptor, generation of water as the only by-product, high substrate selectivity, and reduced environmental burden. These characteristics make laccases attractive tools for sustainable biomass valorization and the development of greener industrial processes.

Phenolic compounds exhibit a well-recognized dual redox behavior in biological systems. Depending on their chemical structure, concentration, oxidation state, and surrounding cellular environment, phenolics may function either as antioxidants or as pro-oxidants. Many lignin-derived phenolics are capable of scavenging free radicals and protecting cells against oxidative damage. However, oxidation of these compounds may also generate reactive intermediates such as semiquinones and quinones that participate in redox cycling, promote reactive oxygen species (ROS) formation, deplete intracellular glutathione reserves, and induce oxidative injury. Consequently, enzymatic transformation of phenolic compounds can substantially alter their biological activity and cellular safety profile. Understanding whether laccase-mediated conversion shifts these compounds toward a more favorable or less favorable redox state is therefore essential before their application in biomedical, nutraceutical, food-contact, packaging, or environmental technologies.

Nevertheless, one of the most significant limitations is that few reports have included information about biological safety, especially about cellular responses to modified products resulting from laccase-mediated catalysis. This is primarily because, during such chemical transformations, oxidation products often include semiquinones or even quinones, whose high reactivity can be reduced by the antioxidant capabilities of the phenolic compounds themselves or of the modified derivatives obtained; alternatively, under specific conditions, redox-cycling quinones could lead to increased cellular oxidative stress. Thus, to determine the real benefits of enzyme-assisted oxidation of bio-based phenolics, there must be comprehensive biological profile studies accompanying any catalytic optimization efforts, in cases targeting biomedical applications, food-contact materials, nutritional additives and even some environmental uses. When the organism comes into contact with more oxidative stress inducers, it will respond by increasing antioxidant defenses such as enzymes which will eliminate reactive species, which can otherwise oxidize biomolecules like proteins, lipids and nucleic acids and reduce the MDA (a marker of oxidative damage by lipid peroxidation) levels, as well as decreasing SOD, CAT, GPx and GSH (a marker of cell response to the damage by oxidative stress or of cell ability to maintain the oxidation–reduction state of cell environments). These biomarkers remain essential for evaluating oxidative injury and protection in living organisms [7,8].

Despite growing interest in laccase-mediated valorization of biomass-derived phenolics, information regarding the cellular safety and redox consequences of the resulting conversion products remains limited. Most previous studies have focused on catalytic efficiency, substrate conversion, antioxidant capacity measured by chemical assays, or material properties, whereas systematic evaluation of cellular oxidative stress responses following enzymatic transformation has received considerably less attention. Therefore, the present study investigated the biocompatibility and oxidative stress profiles of laccase-converted products derived from representative lignin-based phenolics, including vanillin, ferulic acid, syringaldehyde, and guaiacol. A system of laccase enzyme (e.g., trametes hirsuta laccase) will be used to oxidize phenolics at mild reaction conditions in aqueous media. The parent phenolics and their oxidized products after treatment with laccase will be exposed to mammalian cells, alongside untreated phenolics, laccase controls (enzyme in water) and positive controls (e.g., hydrogen peroxide or tert-butyl hydroperoxide). The effects will be quantified by assessing cell viability and by measuring MDA, SOD, CAT, GPx and GSH via appropriate ELISA assay kits. This approach will allow for comparisons between untreated substrates, laccase-treated products and other related controls in terms of cellular biocompatibility and oxidative stress induction. Through this study, we aim to address a relevant translational question concerning the safety and efficacy of laccase-converted bio-phenolics in terms of their potential for improving health outcomes, supporting the circular bioeconomy and paving the way for novel biotechnological solutions based on enzyme-assisted green oxidation of biomass-derived chemicals and functional biomaterials for various sustainable applications.

## 2. Materials and Methods

### 2.1. Chemicals and Reagents

Biomass-derived phenolic compounds including vanillin (Cat. No. V2375), ferulic acid (Cat. No. 128708), syringaldehyde (Cat. No. S7602), and guaiacol (Cat. No. W253200), each with a purity of ≥98%, were purchased from Sigma-Aldrich (St. Louis, MO, USA). The phenolic substrates were used at an initial concentration of 2.0 mM for laccase-catalyzed conversion experiments. Laccase from *Trametes versicolor* (Cat. No. 38429, Sigma-Aldrich, St. Louis, MO, USA; specific activity ≥ 0.5 U/mg) was used as the biocatalyst. ABTS (Cat. No. A1888, Sigma-Aldrich) was employed for determination of laccase activity. Dulbecco’s Modified Eagle Medium (DMEM), fetal bovine serum (FBS), penicillin–streptomycin solution, phosphate-buffered saline (PBS), trypsin–EDTA, and other cell culture reagents were obtained from HiMedia Laboratories (Mumbai, India). Commercial assay kits for the determination of malondialdehyde (MDA; Cat. No. A74477, Antibodies.com, Cambridge, UK), superoxide dismutase (SOD; Cat. No. CS0009, Sigma-Aldrich, St. Louis, MO, USA), catalase (CAT; Cat. No. 11363727001, Merck, Darmstadt, Germany), glutathione peroxidase (GPx; Cat. No. MAK437, Merck/Sigma-Aldrich, St. Louis, MO, USA), and reduced glutathione (GSH; Cat. No. MAK440, Merck/Sigma-Aldrich, St. Louis, MO, USA) were used according to the manufacturers’ instructions.

### 2.2. Preparation of Laccase-Catalyzed Phenolic Conversion Products

For laccase-catalyzed oxidation, vanillin, ferulic acid, syringaldehyde, and guaiacol were prepared at an initial concentration of 2.0 mM in aqueous sodium acetate buffer. The reactions were carried out in 50 mM sodium acetate buffer (pH 5.0), a condition commonly employed for fungal laccase activity, using laccase from *Trametes versicolor* as the biocatalyst. Reaction mixtures were incubated for 6 h at 30 °C under atmospheric oxygen conditions with gentle agitation to ensure adequate oxygen availability for enzymatic oxidation. Untreated phenolic substrate controls, laccase-only controls, buffer-only negative controls, and oxidative stress positive controls were processed under identical conditions.

**Figure 1:**
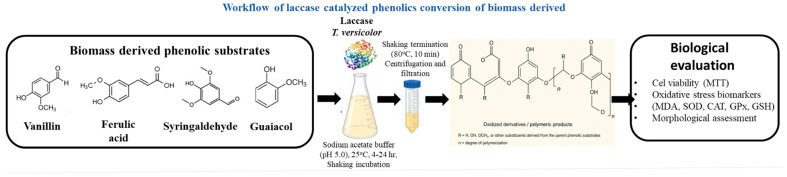
Experimental workflow for laccase-mediated conversion of biomass-derived phenolic compounds and subsequent biological evaluation. Biomass-derived phenolic substrates (vanillin, ferulic acid, syringaldehyde, and guaiacol) were subjected to laccase-catalyzed oxidation under controlled reaction conditions. Following enzyme inactivation, centrifugation, and filtration, the resulting conversion products were evaluated for cellular biocompatibility using MTT assay, oxidative stress biomarker analysis (MDA, SOD, CAT, GPx, and GSH), and morphological assessment.

Following incubation, reactions were terminated by heat inactivation at 80 °C for 10 min. Insoluble reaction products were removed by centrifugation, and the resulting supernatants were passed through 0.22 µm syringe filters before use in subsequent biological experiments. Prior to cellular exposure, reaction products were diluted in culture medium to the required working concentrations. The selected reaction conditions (50 mM sodium acetate buffer, pH 5.0, 30 °C) were based on previously reported operating conditions commonly used for fungal laccases from the Trametes species, which exhibit optimal activity and stability under mildly acidic conditions [2,3]. The overall experimental workflow used for laccase-mediated conversion of biomass-derived phenolics and subsequent biological evaluation is illustrated in Figure 1.

### 2.3. Confirmation of Laccase Activity

Laccase activity was verified using the ABTS oxidation assay. Briefly, ABTS was prepared in 50 mM sodium acetate buffer (pH 5.0) and incubated with laccase under standard assay conditions. Oxidation of ABTS by laccase was monitored by measuring the increase in absorbance at 420 nm, corresponding to the formation of the ABTS radical cation. Absorbance measurements were recorded using a BioTek 800 TS Absorbance Reader (Agilent Technologies, Santa Clara, CA, USA). One unit (U) of laccase activity was defined as the amount of enzyme required to oxidize 1 µmol of ABTS per minute under the assay conditions. The laccase preparation used in this study was obtained from *Trametes versicolor* (Sigma-Aldrich; specific activity ≥ 0.5 U/mg), and enzyme activity was confirmed prior to use in substrate conversion experiments.

### 2.4. Cell Culture

Human embryonic kidney (HEK-293) cells were obtained from the National Centre for Cell Science (NCCS), Pune, India. The cells were maintained in DMEM with 10% FBS and 1% penicillin–streptomycin under standard culturing conditions at 37 °C under 5% CO_2_ gas atmosphere within a humidified incubator until cell confluency of 80–90% was achieved, at which point they were dissociated using trypsin–EDTA until ready for experimental use. The cell population was used at or below passage 5 for the relevant experiments and were seeded into 96-well culture plates in sufficient numbers to allow them to form monolayers after attachment overnight prior to treatments.

### 2.5. Treatment of Cells with Laccase-Converted Products

For exposure studies, cells were seeded in 96-well plates and allowed to attach overnight prior to treatment. Untreated phenolic compounds, the corresponding laccase-converted products, laccase-only controls, oxidative stress positive controls (H_2_O_2_), and untreated negative controls were prepared in complete DMEM and applied to cells for 24 h. For cell viability experiments, a range of concentrations (200–800 µM) was evaluated. For oxidative stress biomarker analyses, cells were exposed to 200 µM of each phenolic compound or corresponding laccase-converted product for 24 h.

Following treatment, culture supernatants were collected and cells were washed twice with phosphate-buffered saline (PBS). Cells were then lysed using an appropriate cell lysis buffer, and the resulting lysates were clarified by centrifugation. The collected cell lysates were subsequently used for determination of MDA, SOD, CAT, GPx, and GSH levels according to the respective assay protocols.

### 2.6. Cell Viability Assay

Cell viability was assessed using the MTT assay following the treatment procedures. Cells were washed once with PBS and incubated with MTT reagent at a final concentration of 0.5 mg/mL for 3–4 h at 37 °C. The resulting formazan crystals were dissolved in 100 µL DMSO, and absorbance was measured at 570 nm with background correction at 630 nm using a BioTek 800 TS Absorbance Reader (Agilent Technologies, Santa Clara, CA, USA). Cell viability was calculated relative to untreated control cells, which were considered 100% viable.

### 2.7. ELISA-Based Oxidative Stress Biomarker Analysis

Oxidative stress and antioxidant biomarkers were measured using the commercially available assay kits listed in Section 2.1 following the manufacturers’ protocols. Following treatment, cells were washed with phosphate-buffered saline (PBS), harvested, and lysed using cell lysis buffer. The resulting lysates were clarified by centrifugation and used for subsequent biomarker analyses.

Standards and samples were added to assay plates according to the respective kit protocols. Following incubation, washing, and substrate development steps, absorbance was measured using a BioTek 800 TS Absorbance Reader (Agilent Technologies, Santa Clara, CA, USA) at the wavelengths specified by the manufacturers of the respective assay kits. Biomarker values were normalized to total protein content and expressed as nmol/mg protein, U/mg protein, mU/mg protein, or µmol/g protein, as appropriate. Standard calibration curves were generated according to the manufacturers’ instructions and used for quantification of the measured biomarkers.

### 2.8. Morphological Assessment

Cellular morphology was examined using inverted phase-contrast microscopy following treatment with untreated phenolic compounds, laccase-converted products, and control treatments. Morphological changes were evaluated qualitatively after 24 h exposure based on cell shape, adherence, cellular density, rounding, shrinkage, and detachment from the culture surface. Representative images were captured under identical imaging conditions and compared qualitatively among treatment groups. No quantitative image analysis or confluence measurements were performed.

### 2.9. Statistical Analysis

All experiments were performed in triplicate, and data were expressed as mean ± standard deviation (SD). Statistical analyses were carried out using GraphPad Prism version 10.0 and SPSS version 26.0. Comparisons among multiple groups were performed using one-way analysis of variance (ANOVA) followed by Tukey’s post hoc test. A *p*-value < 0.05 was considered statistically significant. Heatmaps and graphical visualizations of oxidative stress biomarkers were generated using GraphPad Prism 11 and R statistical software R 4.6.0. Principal component analysis (PCA) was additionally performed to evaluate clustering patterns among treatment groups based on oxidative stress profiles.

Integrated redox score analysis was performed to provide a composite representation of the overall oxidative stress status across treatment groups. Individual biomarker values (MDA, SOD, CAT, GPx, and GSH) were first standardized using Z-score normalization relative to the untreated control group. Since MDA represents oxidative damage whereas SOD, CAT, GPx, and GSH represent antioxidant defense, antioxidant biomarker scores were directionally adjusted before integration. The integrated redox score was then calculated as the combined normalized contribution of oxidative and antioxidant biomarkers. More negative values indicate greater oxidative imbalance and antioxidant depletion, whereas values closer to zero indicate preservation or restoration of physiological redox homeostasis. The score was used as an exploratory comparative index to facilitate multivariate interpretation of treatment-related oxidative stress patterns and was not intended as a validated clinical biomarker.

## 3. Results

**Figure 2:**
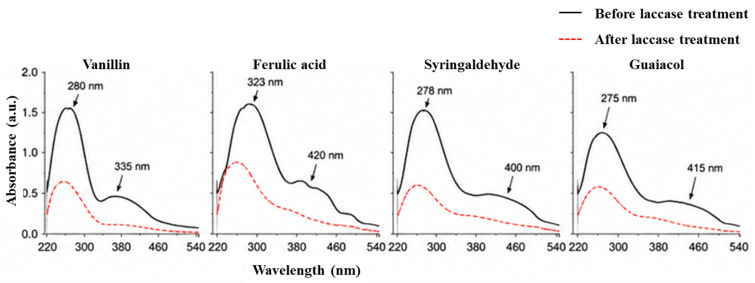
UV–Vis spectral characterization of biomass-derived phenolic substrates before and after laccase-mediated oxidation. UV–Vis absorption spectra of vanillin, ferulic acid, syringaldehyde, and guaiacol before and after treatment with *Trametes versicolor* laccase. Enzymatic oxidation resulted in alterations of characteristic absorption bands, including attenuation of native aromatic peaks and appearance of broader absorbance features, indicating substrate transformation and formation of oxidized derivatives and higher-molecular-weight products.

Laccase-mediated oxidation of all selected biomass-derived phenolic substrates was confirmed by ABTS activity measurements and UV–Vis spectroscopic analysis. Distinct spectral alterations were observed following enzymatic treatment, indicating substrate conversion and formation of oxidation products (Table 1, Figure 2).

A standard ABTS oxidation assay was routinely used to measure steady-state enzyme activity and to monitor stable laccase activity over time. Enhanced absorbance at 420 nm was achieved under reaction conditions that corresponded to maximal laccase enzyme activity. UV-visible spectral scanning was carried out on the same substrates following laccase reaction. The changes in UV-visible spectra confirmed that distinct products were formed by enzymatic activity and that these varied from substrate to substrate. Of the tested substrates, both vanillin and ferulic acid showed the highest apparent conversion with a decrease in substrate absorbance at their native wavelength and an increase in product-derived absorbance. No significant change in UV-visible spectra was noted for the control samples consisting of laccase in buffer or buffer alone (no substrate) throughout the period of reaction, thereby indicating that the noted transformations were mediated by the laccase enzyme and not as a result of autoxidation of the substrate.

UV–Vis analysis demonstrated substantial spectral alterations following laccase treatment of all tested phenolic substrates, indicating enzymatic oxidation and formation of new reaction products. Vanillin and guaiacol exhibited a reduction in the intensity of their native aromatic absorption bands near 275–280 nm, accompanied by the appearance of broader absorbance features consistent with oxidation product formation. Ferulic acid showed attenuation of the characteristic conjugated aromatic peak near 320 nm together with broadening of the absorbance profile, while syringaldehyde displayed loss of native aromatic absorption and the emergence of broad bands in the 350–450 nm region. These spectral changes are consistent with laccase-mediated generation of phenoxy radicals, quinone intermediates, and higher-molecular-weight oxidation products.

These results provide empirical evidence for laccase-catalyzed conversion of common biomass-derived phenolics under the selected reaction conditions and provide the rationale for subsequent examination of the biocompatibility and oxidative stress responses associated with the resulting conversion products.

### 3.1. Effect of Laccase-Converted Phenolic Products on Cell Viability

**Figure 3:**
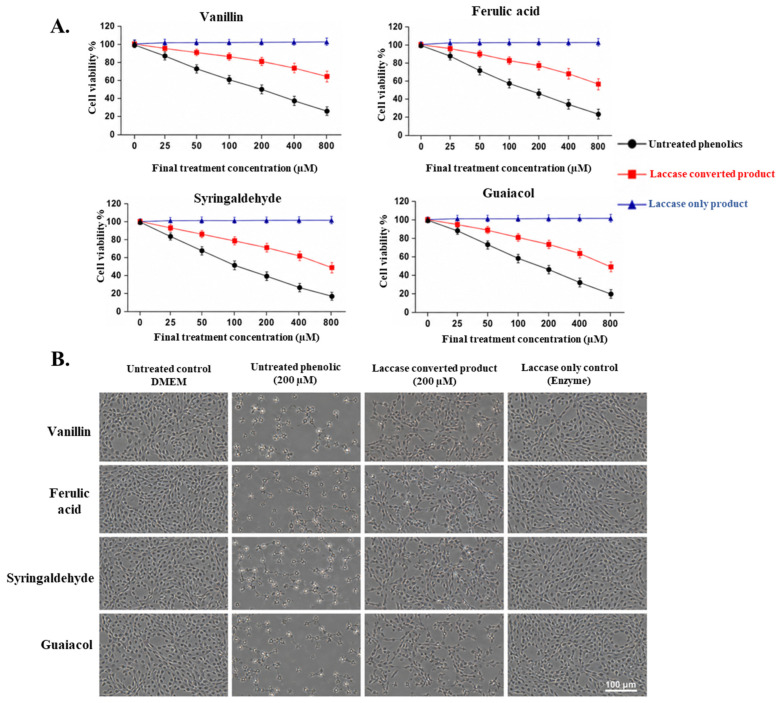
Effects of untreated and laccase-converted biomass-derived phenolic compounds on cellular metabolic activity and morphology. (**A**) MTT assay showing concentration-dependent effects of untreated phenolic compounds and their corresponding laccase-converted products following 24 h exposure. Concentrations shown on the x-axis represent the final concentration of untreated phenolic compounds or equivalent laccase-converted products in the cell culture medium (25–800 µM). Untreated phenolic compounds exhibited progressively reduced MTT reduction activity with increasing concentration, whereas laccase-converted products showed substantially improved cellular tolerance. The laccase-only control demonstrated minimal effects across the tested concentration range. Data are presented as mean ± SD of three independent experiments. (**B**) Representative phase-contrast micrographs of cells following exposure to untreated phenolic compounds (200 µM), corresponding laccase-converted products (200 µM), laccase-only control, and untreated control. Cells exposed to untreated phenolic compounds exhibited reduced cellular density, increased rounding, and loss of normal morphology, whereas cells treated with laccase-converted products displayed improved cellular morphology and adherence, comparable to untreated controls. Scale bar = 100 µm.

The effects of enzymatically processed phenolic products on cellular metabolic activity were evaluated using the MTT assay in HEK-293 cells. Both the untreated phenolic starting materials and their laccase-treated counterparts were introduced to cells for 24 h and cell viability determined by MTT assay. We identified that cellular responses differed markedly between the native phenols and their respective laccase-transformed mixtures. Untreated ferulic acid and syringaldehyde exhibited moderate cell viability reduction when applied at elevated doses in comparison to untreated controls. Conversely, the laccase conversion product mixture of both resulted in markedly improved metabolic activity relative to untreated phenolics, implying reduced toxicity from the phenolic compounds after enzyme oxidation. Vanillin-derived products also exhibited low toxicity and relatively consistent cell survival throughout the range of concentrations tested. Among the studied samples, guaiacol-treated cells suffered from a more extensive reduction in viability after incubation with the untreated phenol, while the guaiacol oxidation product mixture offered a partial rescue. The laccase-only (blank) showed no toxicity to the cell lines, suggesting that the cellular response reported was not a consequence of enzyme activity alone but instead that the phenolic and converted phenols were toxic to cell viability. Regarding dose dependence, all treatment groups shared a similar trend. At higher laccase treatment concentrations, phenolic products appeared to still maintain cell viability at greater than 80%. However, higher doses of products generally led to small-to-modest cell reduction, depending on the phenolic substrate type. Notably, the viability numbers of certain laccase-treated products remained elevated compared to their untreated respective phenol starting material. Morphological observations qualitatively supported the findings obtained from the MTT assay. Cells incubated with native phenolic compounds exhibited morphological features consistent with cellular stress, including reduced cellular density, rounding, slight shrinkage, and partial detachment. In contrast, cells treated with laccase-converted products showed preservation of cellular morphology, improved adherence, and reduced evidence of morphological damage. Based on experiments, laccase-mediated oxidation appears to potentially change the interaction of these bio-derived phenolics with human cells and to create a more benign product (Figure 3) (Supplementary Table S1).

### 3.2. ELISA-Based Oxidative Stress and Antioxidant Biomarker Profiling

**Figure 4:**
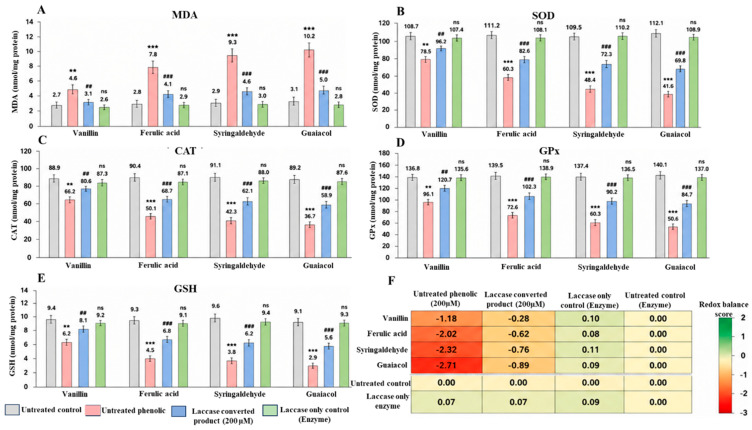
ELISA-based oxidative stress and antioxidant biomarker profiling in HEK-293 cell lysates following 24 h exposure to untreated and laccase-converted phenolic compounds (200 µM). Malondialdehyde (MDA) levels indicating lipid peroxidation and cellular damage are represented (**A**). Exposure to the untreated compounds induced an increase in MDA levels to above the negative control, whereas the treated compounds elicited a significant decrease in lipid peroxidation. The superoxide dismutase (SOD) activity levels are displayed (**B**). Treatment with the untreated compounds led to a reduction in the overall SOD activity level. The treated compounds caused a partial increase in the SOD activity level up to a value close to the control. CAT levels across the treatment groups are shown (**C**). Compared to the untreated phenolic samples, the treated samples maintained a higher proportion of CAT activity. The activity levels of glutathione peroxidase (GPx) are depicted (**D**). Untreated phenolic compounds dramatically lowered GPx levels compared to the negative control. Treatment with the enzymatically modified phenolic products allowed the cells to sustain relatively high GPx levels. The GSH levels in cells treated with the phenolic products over 24 h are plotted (**E**). Treated cells show higher intracellular GSH concentrations compared to cells that were exposed to the untreated compounds. An integrated redox balance heatmap demonstrating the oxidative stress and antioxidant biomarkers across the diverse treatments is presented (**F**). Higher levels of red indicate an increased oxidative imbalance, whereas increased green colors suggest improved redox balance and preservation of antioxidant enzymes. Values are presented as means ± standard deviation (n = 3). Statistical analysis was performed using one-way ANOVA followed by Tukey’s post hoc test. ** *p* < 0.01, and *** *p* < 0.001 vs. untreated control; ## *p* < 0.01 and ### *p* < 0.001 vs. corresponding untreated phenolic group. Untreated phenolic compounds were tested at 200 µM concentration. Units: MDA (nmol/mg protein), SOD and CAT (U/mg protein), GPx (mU/mg protein), and GSH (µmol/g protein).

We performed ELISA-based analysis for these biomarkers to see if laccase-treatment resulted in a less toxic profile regarding oxidative stress caused by those biomass phenolics. Untreated phenolic-treated cells exhibited more damage, levels of MDA increasing, whereas the antioxidants SOD, CAT, GPx and GSH diminished. Of the three non-laccase-treated phenolic products that yielded a stress response, guaiacol generated the strongest one. MDA levels became high and both CAT and GPx activities declined as the other antioxidants were reduced. Ferulic acid increased MDA and lowered the antioxidants, but not to the extent that guaiacol did. Vanillin showed the least amount of stress among the non-treated phenolic treatments. Cells treated with the laccase-converted products of these phenolic compounds did not suffer nearly as much cellular damage; they displayed lower MDA levels and higher antioxidant biomarker levels relative to the corresponding untreated phenolic groups (Figure 3). The laccase-only group provided biomarker data similar to the untreated control, indicating the residual enzyme had no noticeable stress effects at the concentration we used. These results lead us to conclude that laccase oxidation detoxifies certain problematic phenolic substrates while producing conversion products that have less deleterious effects on the redox state of cells (Table 2, Figure 4). ELISA results indicated laccase conversion resulted in decreased oxidation and a healthier redox state within the cell relative to the parent phenolic compounds.

### 3.3. Integrated Redox Balance and Comparative Oxidative Stress Analysis

**Figure 5:**
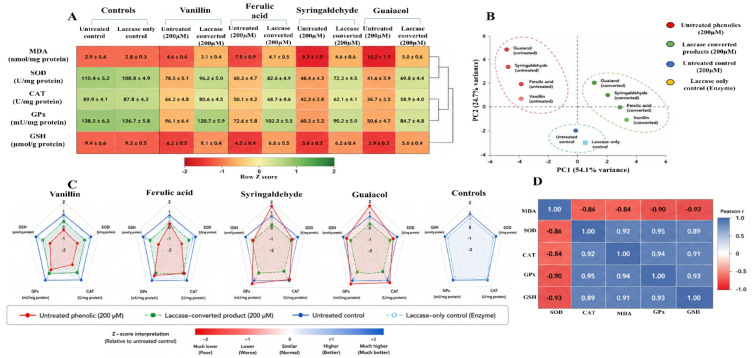
Integrated multivariate oxidative stress profile and redox balance of untreated vs. laccase-converted phenolic products. (**A**) Hierarchical clustering heatmap illustrating oxidative stress markers and antioxidant status patterns within the treatment groups. Intensities in each cell correspond to row-wise Z-score normalized values, where the red intensity denotes increased oxidative stress/lower antioxidant capacity and the green intensity means improved antioxidant status. The untreated phenolic treatment groups were clearly separated from the laccase-converted phenolic product treatment groups and the control groups as shown by distinct clustered groups in the heat map, while the laccase-converted phenolic product treatments also showed partial discrimination from controls. (**B**) Principal component analysis (PCA) score plot with integrated biomarker analysis across samples. Separate clusters of untreated phenolics-treated groups were grouped on the oxidative stress region, whereas the laccase-treated products moved towards control-associated clusters. (**C**) Radar (spider) plot showing integrated oxidative stress profile. Individual phenolic products and control in each group were displayed in radar plots about Z-score normalized data for all the integrated oxidative stress markers. The untreated phenolic product treatment groups indicated high oxidative stress and low antioxidant potential, which was restored by the application of laccase for their treatment toward control. (**D**) Pearson correlation map showing interrelationship between each measured oxidative stress and antioxidant marker. The MDA content had negative correlations with SOD, CAT, GPx and GSH content, while antioxidant markers demonstrated strong positive correlations with each other. Data are presented as mean ± SD (n = 3). Z-score normalization was performed relative to untreated control values. Positive Z-scores indicate higher relative biomarker levels, whereas negative values indicate reduced expression/activity relative to control conditions. MDA: malondialdehyde; SOD: superoxide dismutase; CAT: catalase; GPx: glutathione peroxidase; GSH: reduced glutathione.

This was verified by calculating a combined oxidative status in the host cells by measuring oxidative and antioxidant marker concentrations for both the untreated phenolic compounds and the laccase-converted phenolic derivatives. Phenolic groups are a common characteristic associated with lignocellulosic compounds. Untreated groups formed discrete clusters distinct from laccase-converted compounds. Oxidative stress markers in the form of decreased antioxidant components SOD, CAT, GPx and GSH and increasing MDA were evident in the untreated phenolics-treated groups compared to the untreated controls. Guaiacol and syringaldehyde displayed the highest oxidative stress in the host cells among all tested phenolics and a drastic decrease in antioxidant defense markers. Vanillin displayed the weakest oxidative stress and ferulic acid produced moderate oxidative stress along with some moderate reduction in antioxidants. On the other hand, after conversion of the phenolic groups to their quinone forms by laccase treatment, the oxidative stress decreased. A reduction was measured for total oxidation markers and a restoration of the antioxidants back to control level was shown for most of the substrates after laccase treatment. Similarly, the phenolic derivatives produced after laccase treatment were close to the untreated control or the laccase-only control; in other words, the phenolics after laccase conversion are closer to the original state than untreated phenolics are. The PCA shows clear segregation between the untreated phenolic groups and the groups where laccase-converted products were added based on both biomarker profiles. The highest eigenvalue contribution in terms of the contribution of biomass is from the oxidative stress markers; in particular, this contributes to the first principal component, whereas the antioxidant markers are spread over the other components—in particular, they contribute to the separation in the second principal component. The majority of laccase-converted product groups in terms of PC 1 scores approached untreated control groups, indicating a substantial reduction in cell oxidative stress, while showing signs of a restored pattern back towards untreated control conditions in antioxidants. Laccase enzymatic conversion of the biomass-derived phenolic groups leads to substantial improvement in the host cells’ redox status and improves antioxidant preservation (Supplementary Table S2) (Figure 5).

### 3.4. Correlation Analysis Between Oxidative Stress and Antioxidant Biomarkers

**Figure 6:**
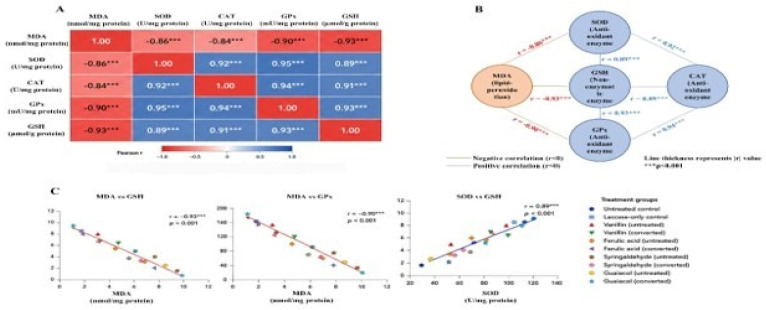
The correlation analysis and biomarker interaction network of oxidative stress parameters. (**A**) Pearson correlation heatmap that examines the correlations between oxidative and antioxidant biomarkers in all treatment groups. MDA negatively correlates strongly with antioxidant biomarkers including SOD, CAT, GPx and GSH. Antioxidant parameters positively correlate strongly among themselves. Correlation coefficients (r values) are shown in the respective cells and red/blue colors represent negative and positive correlations, respectively. (**B**) The biomarker interaction network presenting the comprehensive correlations between the oxidative stress and antioxidant defense systems. MDA (marker of lipid peroxidation) presents strong inverse correlations with antioxidant biomarkers, whereas SOD, CAT, GPx and GSH show consistent positive relationships. Line colors (orange/red: negative correlation; blue: positive correlation) show correlation direction and line thickness represents correlation strength. (**C**) Scatter plots demonstrating key biomarker correlations, including MDA versus GSH, MDA versus GPx and SOD versus GSH. The untreated and phenolic-treated groups are shown clustered towards higher oxidative stress and lower antioxidant capacity, whereas the laccase-treated groups show preserved antioxidant capacity and decreased oxidative stress. Correlation analysis was performed using the Pearson correlation coefficient (r). *** *p* < 0.001. MDA: malondialdehyde; SOD: superoxide dismutase; CAT: catalase; GPx: glutathione peroxidase; GSH: reduced glutathione.

The investigation of the relationship of various parameters was performed using correlation analysis for all monitored oxidative stress biomarkers (MDA, SOD, CAT, GPx and GSH). Lipid peroxidation-associated parameters, on the one hand, exhibited different associations to the antioxidants, on the other hand. Thus, a strong negative association of MDA to SOD, CAT, GPx and GSH was observed, meaning the increase in lipid peroxidation corresponds to a reduction in cellular antioxidants. Between the parameters checked, the tightest negative correlation was determined for MDA to GSH, followed by GPx and SOD. These indicate that the increase in oxidation stress due to untreated phenolic treatment goes hand in hand with the deterioration of the antioxidant potential in cells and reduced cellular redox homeostasis. On the other hand, antioxidant parameters showed a strong positive interrelationship. SOD was positively associated with CAT, GPx and GSH concentrations which suggests the combined activity of antioxidant enzymes against oxidative damage. Also, GPx and GSH exhibited strong positive correlations and are closely linked with their role within the glutathione pathway for detoxification. The correlations, presenting clear differentiation between the markers of oxidative stress and defense: groups without phenolic treatment are located toward high MDA values and low antioxidant statuses, while groups of phenolics treated with laccase are clustered to improve antioxidant correlations and a reduction in stress, represented by MDA. Based on this finding, it is reasonable to confirm that the treatment of biomass phenolic compounds by means of laccase modifies their oxidative characteristics, making them more prone to maintain intracellular antioxidants, reduce oxidative stress and restore redox homeostasis to cells (Figure 6).

Although Pearson correlation analysis identified overall trends among the measured biomarkers, it should be noted that oxidative stress responses are biologically complex and may not follow strictly linear relationships. Antioxidant enzymes such as SOD, CAT, and GPx can exhibit compensatory increases during early oxidative stress, followed by reductions under conditions of prolonged or severe oxidative damage. Therefore, the observed correlations should be interpreted as descriptive associations within the experimental dataset rather than as direct mechanistic relationships.

## 4. Discussion

In our present study, we show that laccase-mediated conversion can transform lignin-derived phenolics, such as vanillin, ferulic acid, syringaldehyde and guaiacol into products with improved compatibility towards cells and a better redox profile. The four selected model substrates (vanillin, ferulic acid, syringaldehyde and guaiacol) represent some of the more abundant lignin-derived aromatics that are pertinent to applications such as those relevant to the biorefinery, the food-packaging, the biomaterial and the bioactive product industry. Numerous literature findings confirm growing interest in such monomers as a viable platform of biorenewable feedstocks for both green chemical syntheses and enzymatic upgrading processes [1,9,10].

It was observable that the absorbance value had increased on the UV-vis spectra of both substrates and colors were formed after incubation with enzyme and substrates as described. This indicates a modification of the molecular structure of phenols as a result of oxidation induced by laccase. The oxidation takes place easily since phenolic structures are readily oxidized to phenoxy radicals on a catalytic and oxidant and the radicals can be combined into larger molecules to form oligomers and polymers. Laccase can be referred to as multi-copper oxidase using O_2_ as sole electron acceptor. Laccase represents a promising, environmentally friendly alternative to common chemical oxidation agents in oxidation reactions, attractive in sustainable bioprocess development. Numerous recent studies on laccase-mediated oxidation and polymerization of phenolics, including phenolic-rich wine lees extracts and the in situ oxidation of hydroxytyrosol to obtain novel bioactivity profiles, support our findings [2,5,6]. The key conclusion of this experiment was that untreated phenolics exhibit cytotoxicity, whereas laccase-formed metabolites cause lesser cytotoxicity. Enzymatic modification by laccase could reduce the direct cytotoxic effect of some phenolic substrates by affecting their properties. Phenolic compounds can exhibit enhanced reactivity and lower solubility through enzymatic oxidation. A report recently documented increased functional, sensory and biological characteristics of the extracts by laccase oxidation as a result of modifying bioactives in lignocellulosic sources and also for the inhibition of bioactivity in polymeric bioactives through oxidation by laccase [11,12,13]. Involvement of oxidative stress was revealed through the results generated by the enzyme-linked immunosorbent assays (ELISA) that can be mechanistically coupled with cell viability. Untreated phenolic extracts increase levels of malondialdehyde (MDA), increase and decrease activities of SOD, CAT, GPx, respectively and decrease the amount of glutathione (GSH). MDA is a marker of oxidative lipid peroxidation, while the enzymatic antioxidants SOD, CAT and GPx protect the organism from cellular damage that occurs from peroxides as well as the nonenzymatic antioxidant GSH. Recently, these biomarkers have been used to characterize the redox balance in cellular system as signs of an oxidative imbalance [14,15].

Of the phenolics identified in the samples, guaiacol and syringaldehyde exhibited more oxidative stress effects than ferulic acid, but less than vanillin. This pattern of effects observed for the identified phenolic components might be related to their structure and functional groups. The pattern is influenced by the number of methoxy groups and aldehyde versus carboxyl functionality, in addition to the capacity of such molecules to generate reactive species that are part of the oxidative process. Vanillin and ferulic acid have been reported in the literature as having both antioxidant as well as anti-inflammatory activities and also act as an enhancer in cancer therapy in some types of human cancer, but their biological activities differ with dose and the context [16,17,18]. The levels of MDA reduced, while SOD, CAT, GPx and GSH levels were partially restored after the conversion of phenolic substances with laccase. As a consequence, these data do not provide convincing evidence that all oxidized products generated by the enzyme possess inherent protective activity, but they do reveal that laccase treatment decreased the pro-oxidant potential of the phenolic substances used under experimental conditions. This is in agreement with a recently published study which showed that the antioxidant properties of both the enzymes and the resulting polymeric materials were affected in differing ways according to the type of phenolic substrates and reaction conditions [5,19,20].

Furthermore, multivariate statistical analyses confirm that untreated phenolic fractions, laccase-converted phenolic compounds and controls differed in terms of all biomarkers. Both hierarchical clustering and principal component analyses show a separation between untreated phenolics as a group from both the laccase-converted fractions and the blank controls. These results reveal that, overall, the entire pattern of biomarker levels differs post-treatment with the enzyme rather than being affected by one specific biomarker. An integrated oxidative score has also shown that the laccase-converted phenolic compounds are closer to blank control values. This type of integrated approach has proved to be more effective because oxidative stress is characterized by the dynamic balance between the rate of damage to cellular components via reactive oxidative species (ROS) and the cell’s defense mechanisms involving antioxidants. Additionally, the correlation matrix shows strong correlations between the studied oxidative and antioxidant biomarkers. There were significant positive associations between antioxidant markers and a negative association between the level of MDA and the levels of SOD, CAT, GPx and GSH. Thus, MDA as an indicator of lipid peroxidation is correlated with reduced antioxidant capacity. Notably, strong positive correlations were obtained between GPx and GSH; therefore, glutathione-based cellular defensive systems represent a critical process for scavenging hydrogen peroxide.

Interpretation of the multivariate analyses should be undertaken with appropriate biological caution. Although MDA, SOD, CAT, GPx, and GSH collectively provide useful information regarding oxidative stress status, these biomarkers represent fundamentally different biological entities. MDA is a downstream product of lipid peroxidation and GSH is a tightly regulated intracellular metabolite, whereas SOD, CAT, and GPx are enzymatic antioxidant defense systems whose functional contributions depend not only on protein abundance but also on catalytic activity, post-translational regulation, substrate availability, and cellular compartmentalization. Consequently, the clustering, correlation, PCA, and integrated redox analyses presented in this study should be viewed as exploratory approaches for summarizing global biomarker patterns rather than as direct representations of the mechanistic interactions governing cellular redox homeostasis.

It should also be recognized that cellular redox regulation is inherently dynamic and frequently involves nonlinear biological responses. Antioxidant enzymes may initially become upregulated as part of adaptive cellular defense mechanisms and subsequently decline when oxidative injury exceeds cellular compensatory capacity. Consequently, the Pearson correlation analyses presented in this study should be viewed as exploratory tools for identifying overall biomarker associations rather than definitive representations of the underlying biological mechanisms. Furthermore, the present study quantified biomarker levels but did not directly evaluate enzyme kinetic activity, post-translational regulation, or other factors that may influence functional antioxidant capacity.

From a green catalysis point of view, these results are crucial for assessing and broadening the applications of biomass-derived phenolics. Biomass derivatives have been studied mostly for conversion efficiencies, chemical radical-scavenging assays or even material properties. However, detailed profiling of their impact on cellular redox status is rare. In line with the major reviews published on laccases and their enzymes’ potential for lignin valorization, and conversion of phenolics towards sustainability and biorefinery applications, cell biological activity, including safety aspects, is often an overlooked issue in linking enzyme conversion research to its industrial application [4,21,22]. Our work contributes a basic cellular safety evaluation of laccase-mediated conversion of selected plant phenolics and can potentially guide a wider use of these enzymes in areas such as functional packaging, food fortification, nutraceutical carriers, biomaterials, drug coatings and other applications where the products could pose environmental risks. Indeed, lignin-derived phenols and modified lignins have emerged as promising alternatives for food preservation, antimicrobial materials, packaging technologies and biomaterials due to their radical-scavenging and antioxidant functionalities [10,13,23].

Nevertheless, the study has a few limitations. First, whereas ELISA-based biomarkers capture reactive oxygen species-level functionality redox parameters they fail to pinpoint oxidation by-products generated by enzymes; therefore, to delineate oxidation by-products, the extent of oligomers and polymerization patterns of oxidized phenolics, future work should also include techniques such as LC-MS/MS, FTIR, NMR and GPC analysis. Furthermore, to establish more biologically meaningful conclusions about the cell viability effects of long-term laccase exposure, additional cell lines, longer exposure protocols and an analysis of inflammatory cytokines must be employed. The MTT assay primarily reflects mitochondrial metabolic activity rather than direct cell viability; therefore, additional assays such as live/dead staining, membrane integrity assays, or apoptosis measurements would further strengthen the biological interpretation of the observed effects. In addition, the morphological assessment performed in this study was qualitative in nature. Future studies incorporating quantitative image analysis, automated confluence measurements, and cell counting approaches would provide a more objective evaluation of cellular responses. In addition, the correlation analyses performed in this study were based on linear statistical relationships and may not fully capture the complex nonlinear dynamics that characterize cellular oxidative stress responses. Future studies incorporating larger datasets, enzyme activity measurements, and nonlinear modeling approaches may provide additional mechanistic insights into redox regulation following laccase-mediated phenolic conversion. The bioactivity of the oxidation by-products and the extent of the oxidation of phenolic compounds vary with laccase dose, pH, the specific mediator, availability of O_2_ and incubation time. Furthermore, the oxidative stress biomarkers evaluated in this study represent biologically distinct processes and were integrated primarily for exploratory multivariate analyses. The ELISA-based measurements primarily reflect biomarker abundance rather than direct enzymatic activity. Future investigations incorporating enzyme activity assays, reactive oxygen species measurements, glutathione cycle dynamics, and mechanistic pathway analyses would provide a more comprehensive understanding of the functional regulation of cellular redox homeostasis following laccase-mediated phenolic conversion.

The results of this study lead us to conclude that laccase may not just serve as a sustainable catalytic method of valorization of biomass-derived phenolic compounds but may also have positive effects on their compatibility and redox cellular impact. A holistic study involving enzyme conversion, testing cell viability, ELISA analysis of the levels of reactive oxygen species (ROS) and multivariate redox analysis may well become a viable pathway in testing bio-derived materials produced via enzyme catalysis. The strategy developed in this study helps provide a more complete characterization of laccase-derived products than just the extent of chemical conversion and thereby opens opportunities for safe production and use of laccase-derived phenolics in the bio-based, biomedical, chemical and packaging sectors.

## 5. Conclusions

The laccase-catalyzed oxidation of biomass-derived phenolics to better suited bioactive and pro-oxidant properties is demonstrated. Vanillin, ferulic acid, syringaldehyde and guaiacol were oxidized by laccase and the resulting products showed less toxicity and oxidative potential compared with their original phenolic substrates. Unmodified phenolic compounds induce dose-dependent cytotoxicity, accompanied by enhanced lipid peroxidation, along with a decrease in antioxidants such as SOD, CAT, GPx and GSH, whereas their oxidized products lead to lower MDA content, partly increase antioxidants and improve the redox balance of cells compared with original untreated substrates. Analysis by hierarchical clustering, PCA, radar plots and correlation revealed that laccase oxidation converts phenolic products towards the antioxidant characteristics observed for the control. These observations therefore suggest the application of laccase in a sustainable and biologically superior modification pathway for biomass-derived phenolics, where biocompatibility and anti-oxidative characteristics can be crucial for successful green bioengineering. With these combined cellular analyses, multivariate redox profiles and catalytic results, we provide a methodology framework which would help the design of safer lignin-derived bioactive materials, antioxidant agents, green packaging systems and environmentally benign bio-products, all contributing to a greener future. Advanced research covering detailed structural identification and longer-term biological studies, as well as clinical evaluations, are still necessary to completely explain the transformation mechanisms and the practical significance of these laccase-derived phenolic transformation products.

## Figures and Tables

**Table 1:** Biomass-derived phenolic substrates used for laccase-catalyzed oxidation and their UV–Vis spectral characteristics following enzymatic conversion. (* *p* < 0.05).

Phenolic Substrate	Biomass/Lignin Source	Major Functional Groups	Initial Concentration (mM)	Reaction Time (h)	Relative Conversion Efficiency *	Major UV–Vis Spectral Change
Vanillin	Lignin-derived aromatic aldehyde	Aldehyde, methoxy, phenolic hydroxyl	2.0	6	Moderate to high	Reduction in ~280 nm aromatic peak with appearance of broad oxidation band
Ferulic acid	Plant cell wall lignin/hemicellulose	Carboxylic acid, methoxy, phenolic hydroxyl	2.0	6	High	Decrease in conjugated peak near ~320 nm and formation of broad oxidized-product spectrum
Syringaldehyde	Hardwood lignin-derived phenolic aldehyde	Aldehyde, two methoxy groups, phenolic hydroxyl	2.0	6	High	Loss of native aromatic absorption and broadening in 350–450 nm region
Guaiacol	Lignin pyrolysis product	Methoxy, phenolic hydroxyl	2.0	6	Moderate	Reduction in ~275 nm peak with gradual broad-band formation
Laccase-only control	—	Enzyme only	—	6	None	No major spectral alteration
Buffer-only control	—	Sodium acetate buffer	—	6	None	Stable baseline spectrum

**Table 2:** ELISA-based oxidative stress and antioxidant biomarker levels in HEK-293 cell lysates following 24 h exposure to untreated and laccase-converted phenolic compounds (200 µM).

Treatment Group	MDA (nmol/mg Protein)	SOD (U/mg Protein)	CAT (U/mg Protein)	GPx (mU/mg Protein)	GSH (µmol/g Protein)	Overall Redox Interpretation
Untreated control	2.9 ± 0.4	110.4 ± 5.2	89.9 ± 4.1	138.5 ± 6.3	9.4 ± 0.6	Normal physiological redox balance
Laccase-only control	2.8 ± 0.3	108.8 ± 4.9	87.8 ± 4.3	136.7 ± 5.8	9.2 ± 0.5	Comparable to untreated control
Vanillin (untreated)	4.6 ± 0.6 **	78.5 ± 5.1 **	66.2 ± 4.8 **	96.1 ± 6.4 **	6.2 ± 0.5 **	Mild oxidative stress induction
Vanillin (laccase-converted)	3.1 ± 0.4 ##	96.2 ± 5.0 ##	80.6 ± 4.5 ##	120.7 ± 5.9 ##	8.1 ± 0.4 ##	Improved antioxidant preservation
Ferulic acid (untreated)	7.8 ± 0.9 ***	60.3 ± 4.7 ***	50.1 ± 4.2 ***	72.6 ± 5.8 ***	4.5 ± 0.4 ***	Moderate oxidative imbalance
Ferulic acid (laccase-converted)	4.1 ± 0.5 ###	82.6 ± 4.9 ###	68.7 ± 4.6 ###	102.3 ± 5.5 ###	6.8 ± 0.5 ###	Reduced oxidative burden
Syringaldehyde (untreated)	9.3 ± 1.0 ***	48.4 ± 4.3 ***	42.3 ± 3.8 ***	60.3 ± 5.2 ***	3.8 ± 0.3 ***	Marked oxidative stress response
Syringaldehyde (laccase-converted)	4.6 ± 0.6 ###	72.3 ± 4.5 ###	62.1 ± 4.1 ###	90.2 ± 5.0 ###	6.2 ± 0.4 ###	Partial restoration of redox balance
Guaiacol (untreated)	10.2 ± 1.1 ***	41.6 ± 3.9 ***	36.7 ± 3.5 ***	50.6 ± 4.7 ***	2.9 ± 0.3 ***	Severe oxidative stress induction
Guaiacol (laccase-converted)	5.0 ± 0.6 ###	69.8 ± 4.4 ###	58.9 ± 4.0 ###	84.7 ± 4.8 ###	5.6 ± 0.4 ###	Improved redox homeostasis following enzymatic conversion

Data are presented as mean ± SD (n = 3). Statistical analysis was performed using one-way ANOVA followed by Tukey’s post hoc test. ** *p* < 0.01 and *** *p* < 0.001 vs. untreated control. ## *p* < 0.01 and ### *p* < 0.001 vs. corresponding untreated phenolic group. Abbreviations: MDA, malondialdehyde; SOD, superoxide dismutase; CAT, catalase; GPx, glutathione peroxidase; GSH, reduced glutathione.

## Data Availability

The original contributions presented in this study are included in the article. Further inquiries can be directed to the corresponding author.

## Supplementary Materials

The following supporting information can be downloaded at: https://www.mdpi.com/article/10.3390/toxics14070550/s1, Table S1: MTT-based cellular metabolic activity of HEK-293 cells following exposure to untreated and laccase-converted phenolic compounds after 24 h exposure; Table S2: Integrated oxidative stress index and multivariate clustering characteristics of untreated and laccase-converted phenolic products.
