# Supplementary material for: Biocompatibility and Oxidative Stress Profiling of Laccase-Catalyzed Conversion Products of Biomass-Derived Phenolics
Source: Toxics. 2026 Jun 24;14(7):550. doi: 10.3390/toxics14070550 (PMC13418121; doi:10.3390/toxics14070550)
Supplement: Supplementary file 1 [file toxics-14-00550-s001.zip › toxics-4357952-supplementary.pdf]

Supplementary Table S1. MTT-based cellular metabolic activity of HEK-293 cells following exposure to untreated and laccase-converted phenolic compounds after 24 h exposure

Treatment group	Concentration (µM)	Cell viability (%) Mean ± SD	Relative cytotoxicity (%)	Morphological observations	Statistical significance
Untreated control	—	100.0 ± 4.2	0.0	Normal spindle morphology with intact adherence and confluency	Reference
Laccase-only control	800	98.2 ± 3.8	1.8	Morphology comparable to untreated control	NS
Vanillin (untreated)	200	71.7 ± 4.9	28.3	Mild cell rounding and reduced confluency	*$p < 0.05$ vs control
Vanillin (laccase-converted)	200	88.7 ± 4.1	11.3	Preserved morphology with minimal rounding	##$p < 0.01$ vs untreated vanillin
Ferulic acid (untreated)	200	52.8 ± 5.3	47.2	Moderate shrinkage, partial detachment, reduced adherence	***$p < 0.001$ vs control
Ferulic acid (laccase-converted)	200	77.4 ± 4.7	22.6	Improved cellular integrity and moderate preservation of confluency	###$p < 0.001$ vs untreated ferulic acid
Syringaldehyde (untreated)	200	55.3 ± 4.8	44.7	Rounded cells with visible reduction in cell density	***$p < 0.001$ vs control
Syringaldehyde (laccase-converted)	200	82.1 ± 4.4	17.9	Comparatively preserved morphology and adherence	###$p < 0.001$ vs untreated syringaldehyde
Guaiacol (untreated)	200	45.2 ± 5.1	54.8	Significant cell rounding and partial detachment	***$p < 0.001$ vs control
Guaiacol (laccase-converted)	200	75.2 ± 4.9	24.8	Improved morphology with moderate restoration of confluency	###$p < 0.001$ vs untreated guaiacol
Vanillin (untreated)	800	25.6 ± 5.8	74.4	Severe reduction in viable adherent cells	***$p < 0.001$ vs control
Vanillin (laccase-converted)	800	64.1 ± 5.0	35.9	Moderate cytotoxicity with partial preservation of morphology	###$p < 0.001$ vs untreated vanillin
Ferulic acid (untreated)	800	22.7 ± 5.9	77.3	Extensive cell detachment and shrinkage	***$p < 0.001$ vs control
Ferulic acid (laccase-converted)	800	57.8 ± 5.3	42.2	Reduced cytotoxicity compared with untreated substrate	###$p < 0.001$ vs untreated ferulic acid
Syringaldehyde (untreated)	800	17.3 ± 4.6	82.7	Marked loss of cellular adherence and integrity	***$p < 0.001$ vs control
Syringaldehyde (laccase-converted)	800	49.6 ± 5.4	50.4	Partial recovery of morphology and viability	###$p < 0.001$ vs untreated syringaldehyde
Guaiacol (untreated)	800	19.8 ± 5.1	80.2	Severe morphological deterioration and low confluency	***$p < 0.001$ vs control
Guaiacol (laccase-converted)	800	50.1 ± 5.0	49.9	Moderate preservation of cellular structure	###$p < 0.001$ vs untreated guaiacol

Abbreviations: NS, not significant. Relative cytotoxicity (%) = 100 − cell viability (%). Data represent mean ± standard deviation from three independent experiments.

Supplementary Table S2: Integrated oxidative stress index and multivariate clustering characteristics of untreated and laccase-converted phenolic products

Treatment group	Integrated redox score*	Oxidative burden ranking	PCA cluster association	Dominant oxidative stress pattern	Overall interpretation
Untreated control	0.00 ± 0.05	Lowest	Control cluster	Physiological antioxidant balance	Normal cellular redox homeostasis
Laccase-only control	0.09 ± 0.04	Lowest	Control-like cluster	Minimal oxidative perturbation	Comparable to untreated control
Vanillin (untreated)	−1.18 ± 0.12	Mild	Oxidative stress cluster	Mild increase in lipid peroxidation with partial antioxidant depletion	Mild oxidative stress induction
Vanillin (laccase-converted)	−0.28 ± 0.08	Low	Intermediate/control-shifted cluster	Improved antioxidant preservation and reduced oxidative damage	Partial restoration of redox balance
Ferulic acid (untreated)	−2.02 ± 0.18	Moderate	Oxidative stress cluster	Elevated MDA with substantial reduction in antioxidant enzyme activity	Moderate oxidative imbalance
Ferulic acid (laccase-converted)	−0.62 ± 0.10	Mild	Intermediate cluster	Reduced oxidative burden compared with untreated substrate	Improved antioxidant status
Syringaldehyde (untreated)	−2.32 ± 0.21	High	Severe oxidative stress cluster	Marked lipid peroxidation and antioxidant depletion	Significant oxidative stress response
Syringaldehyde (laccase-converted)	−0.76 ± 0.11	Mild to moderate	Intermediate cluster	Partial restoration of cellular antioxidant defense	Reduced oxidative injury
Guaiacol (untreated)	−2.71 ± 0.24	Highest	Severe oxidative stress cluster	Severe oxidative imbalance with pronounced antioxidant depletion	Strong oxidative stress induction
Guaiacol (laccase-converted)	−0.89 ± 0.13	Moderate	Intermediate cluster	Improved redox balance relative to untreated guaiacol	Attenuated oxidative toxicity

*Integrated redox score was calculated using normalized Z-score integration of composite biomarker profile and antioxidant biomarkers (MDA, SOD, CAT, GPx, and GSH). Integrated redox score represents an exploratory composite index derived from Z-score normalized biomarker measurements (MDA, SOD, CAT, GPx, and GSH). Because these biomarkers reflect distinct biological processes, the score should be interpreted as a descriptive summary of overall biomarker patterns rather than as a direct quantitative measure of cellular redox homeostasis.

Abbreviations: PCA, principal component analysis; MDA, malondialdehyde; SOD, superoxide dismutase; CAT, catalase; GPx, glutathione peroxidase; GSH, reduced glutathione.
